# Prevalence and associated factors of burnout among working adults in Southeast Asia: results from a public health assessment

**DOI:** 10.3389/fpubh.2024.1326227

**Published:** 2024-03-14

**Authors:** Amani Fadzlina Abdul Aziz, Tiffanie Ong

**Affiliations:** Naluri Hidup Sdn Bhd, Kuala Lumpur, Malaysia

**Keywords:** burnout, employee mental Health, employee burnout, Southeast Asia (SEA), mental health

## Abstract

The COVID-19 pandemic has spotlighted the mental health crisis among employees worldwide. However, burnout research is often industry- or occupation-specific, and limited knowledge currently exists on the prevalence of burnout in the general working population of Southeast Asia. This study aims to examine the prevalence of employee burnout and its associated factors among working adults in Southeast Asia using secondary data. 4,338 full-time employees aged 18–65 years old living in Malaysia, Singapore, Philippines, and Indonesia were assessed for burnout, depression, anxiety, stress, and sociodemographic characteristics as part of an online public health assessment in October 2022. The prevalence of burnout in the region was 62.91%. Burnout was highest among employees in the Philippines (70.71%) and lowest in Malaysia (58.13%). Experiencing burnout was associated with severe or extremely severe depression (AOR = 6.48 [95% CI = 5.06–8.33]), anxiety (AOR = 2.22 [1.74–2.85]), and stress (AOR = 5.51 [4.13–7.39]). Working more than 50 hours a week (AOR = 1.38 [1.04–1.82]) and being very dissatisfied with the job led to higher odds of burnout (AOR = 16.46 [8.99–30.53]). Alarmingly, more than half of working adults in the region are reporting increased levels of burnout, and improving employee mental health and work conditions may be key to improving employee burnout in the region. Findings contribute to existing research on burnout prevalence in the region and provide more comprehensive insights into understanding the factors driving employee burnout in the working population of Southeast Asia 2 years after the onset of the pandemic.

## 1 Introduction

The COVID-19 pandemic has led to an unprecedented rise in employee burnout worldwide (1, 2), as the global workforce faces major changes in work norms and practices in the short span of 3 years (3–5). Defined as a work-related state of exhaustion, burnout is characterized by extreme tiredness or fatigue, an impaired ability to regulate cognitive and emotional processes, and mental distancing (6). Specific to the occupational context (7), burnout corresponds to prolonged and chronic workplace stress rather than occasional one-off stressors (8, 9), and under the Job Demands-Resources (JDR) theory, is thought to result from an imbalance between work demands and employee resources (10). When left unaddressed, burnout can lead to adverse health consequences for individuals and can translate into a substantial economic burden to employers as it facilitates absenteeism, presenteeism, counterproductive organizational behaviors, increased turnover intentions, and reductions in work performance (10).

Although burnout was initially studied within the context of healthcare workers, it has now been established that burnout can occur across most occupational groups, though professions that involve constant demands and emotional labor tend to be disproportionately affected (8). Demographic variables such as age, gender, and marital status have also been studied in relation to the development of burnout, though findings have mostly been inconclusive with regard to which groups are more vulnerable to burnout (10–13). Separately, work-related factors such as working hours (14), emotional labor (15), workload (16), and job dissatisfaction (17), are known to directly correlate with burnout. Despite burnout being an entirely separate and distinct phenomenon (18), symptomatic overlapping can occur between burnout and other forms of mental illness (19), with existing research showing burnout to correlate with symptoms of depression, anxiety, and stress (5, 20).

Existing research on the prevalence of employee burnout is often centered around employees in the healthcare industry. Woo et al. reported a global burnout prevalence of 11.23% among nurses across 49 countries (21), while the global prevalence of burnout among general practitioners was estimated at 37% (22). In Southeast Asia, a pooled regional prevalence of burnout among gastroenterologists has been estimated at 17.1%, with inter-country variations identifying Malaysia, Singapore, and Brunei as countries with a burnout prevalence rate exceeding 30% (23). However, these prevalence rates only reflect that of healthcare workers’ burnout and do not represent the prevalence of employee burnout in the general working population. Given the short- and long-term effects of the COVID-19 pandemic on employee well-being worldwide (24), it is critical to attain a comprehensive understanding of the phenomenon of employee burnout, irrespective of occupation and industry.

To our knowledge, there is insufficient evidence on the prevalence of employee burnout among the general working population of Southeast Asia. Given that unmanaged burnout leads to adverse psychological, behavioral, health, and economic consequences to both individuals and organizations (10, 25), it is crucial to understand the full extent of the phenomenon in the region to guide future intervention or prevention efforts. Hence, the primary objectives of this study are to determine the prevalence of employee burnout among full-time working adults in Southeast Asia and to identify the associated factors that contribute to the development of burnout among working adults in the region. As a secondary objective, this study also looks into the prevalence of depression, anxiety, and stress among working adults in Southeast Asia.

## 2 Materials and methods

### 2.1 Study design and procedures

This cross-sectional epidemiological retrospective study uses secondary data collected as part of an annual public mental health assessment conducted by Naluri Hidup Sdn Bhd (Naluri), in conjunction with a month-long Mental Health Awareness Campaign. Throughout October 2022, respondents were recruited through convenience sampling via paid advertising on Naluri’s social media channels (e.g., Facebook, LinkedIn, Instagram) and advertising platforms (e.g., Google). Respondents who were interested in the mental health assessment were directed to an online questionnaire hosted at www.naluri.life. The mental health assessment questionnaire was divided into three sections in the following order: (1) psychological distress; (2) burnout, and; (3) optional sociodemographic questions. The landing page of the assessment displayed instructions on how to complete the assessment, as well as information on the nature and purpose of the mental health assessment.

### 2.2 Ethical consideration

By proceeding with the assessment, participants provided implied consent by accepting and agreeing with Naluri’s data policy, which includes a clause stating that their anonymised data may be used for research purposes. Ethics approval for this study was obtained from the Medical Research & Ethics Committee, Ministry of Health Malaysia (NMRR ID-22-02193-GDR). Although this study was planned prior to data collection, ethics approval was only obtained toward the end of the data collection period, which led to changing the study’s design from prospective to retrospective. No personally identifiable information was collected and all data was obtained anonymously and handled confidentially. Participants did not receive any tokens or incentives as part of participation in the study. In line with the EQUATOR Network reporting guidelines, a complete STROBE checklist for this study is provided (Supplementary Table S1).

### 2.3 Study participants

Participants of this study were respondents of the mental health assessment who fulfilled the study inclusion criteria, which were set to full-time employed adults aged 18–65 years old living in Southeast Asia, specifically in Malaysia, Singapore, Philippines, and Indonesia, who had completed the English-language version of the survey on Naluri’s website. A convenience sampling strategy was employed to select only respondents who fulfilled the pre-specified inclusion criteria out of all the responses from the mental health assessment. Respondents who were outside of the target age range, did not hold full-time employment, resided outside of the target countries, and completed the assessment in a local non-English language were excluded. Although the mental health assessment was available in multiple languages, setting the inclusion criteria to those who completed the assessment in English was done to optimize the study’s validity as the instruments used in the mental health assessment were validated in English. Additionally, the mental health assessment was designed to allow respondents to skip sociodemographic questions in order to encourage as many respondents to complete the assessment as possible. Hence, only complete responses across all sections of the assessment were included in the study. Our initial protocol was specified to include responses from residents in Thailand, with a minimum sample size of *n* = 384 required based on an estimated prevalence of 49.3% and a precision of 5% (26–28). However, as only *n* = 44 responses from Thailand fulfilled the inclusion criteria, we elected to remove responses from Thailand from our final analysis as a small sample size would have resulted in inaccurate and imprecise estimates (29, 30).

### 2.4 Measures and instruments

#### 2.4.1 Burnout assessment tool (BAT-12)

Burnout was measured using the work version of the 12-item Burnout Assessment Tool (BAT-12), a validated short-version of the BAT that measures four core symptoms of burnout - exhaustion, mental distance, cognitive impairment, and emotional impairment (6, 31, 32). The work version of the BAT-12 was chosen due to its applicability across all forms of work and professions, and for its ability to classify burnout along a continuum of “low” to “very high,” which has been recommended as a superior way of measuring burnout (33). In addition, the BAT-12 was also preferred for its ability to provide a composite score that comprehensively reflects the overall experience of burnout, as opposed to more traditional burnout measurements, like the Maslach Burnout Inventory, which was developed primarily as an instrument to detect the different dimensions of burnout (9). Items are scored on a 5-point scale from 1 - “never” to 5 - “always,” and a total composite burnout score is obtained by averaging the sum of all 12 items (6). Burnout scores were classified as Low, Average, High, and Very High using the more conservative cut-offs of Low = 1.00–1.50; Average = 1.51–2.79; High = 2.80–3.66; Very High = 3.67–5.00 (6). The use of more conservative cut-off scores is intended to control for possible cross-cultural bias, as previous cross-cultural research revealed that Asian populations tend to score higher in the BAT compared to Western populations (34). The presence of burnout was defined as recording ‘High’ or ‘Very High’ levels of burnout based on the BAT-12. The BAT has previously been validated for cross-cultural and online use (34), and its convergent validity against traditional burnout measures such as the Maslach Burnout Inventory has been established (35). The internal reliability of the BAT-12 for this study is α = 0.94.

#### 2.4.2 Depression, anxiety, stress scales (DASS-21)

Psychological distress was measured using the DASS-21, a set of self-report scales comprising 21 items equally divided into three subscales measuring the emotional states of depression, anxiety, and stress (36). Items are scored on a 4-point Likert scale ranging from 0 – “did not apply to me at all” to 4 – “applied to me very much or most of the time,” and final scale scores are obtained by multiplying subscale scores by 2, with higher scores indicating higher severity for each scale. Cutoff scores for each subscale are used to further classify the scores into conventional severity labels - Normal, Mild, Moderate, Severe, and Extremely Severe (36). The presence of depression, anxiety, and stress was, respectively, defined as recording ‘Severe’ or ‘Extremely Severe’ levels of each domain based on scores of the DASS-21.

#### 2.4.3 Sociodemographic questions

The exposure variables were measured using sociodemographic questions on demographic and work-related characteristics. Specifically, participants were asked to supply their year of birth, gender, country of residence, relationship status, employment status, work industry, job seniority, the average number of hours they worked per week (inclusive of overtime), current working setup (i.e., in-office, remote, hybrid), and job satisfaction. Job satisfaction was assessed using a single-item measure, “Taking everything into consideration, how do you feel about your job as a whole?,” rated on a seven-point Likert scale (1 = extremely dissatisfied, 7 = extremely satisfied) with higher scores indicating higher levels of job satisfaction, which has been shown to be valid and reliable in assessing job satisfaction among employees (37). The sociodemographic questions were not compulsory for the respondents to complete.

### 2.5 Statistical analysis

All analyses were performed on RStudio version 2022.07.0 + 548, using R version 4.2.1. Statistical tests performed were 2-sided and evaluated at a *p* < 0.05 significance threshold. The prevalence of burnout, depression, anxiety, and stress were reported with their respective 95% confidence intervals (CIs). In addition, means and standard deviations for burnout, depression, anxiety, and stress scores were reported for the overall sample, as well as for each sociodemographic group (Supplementary Table S2).

Simple logistic regressions were performed to investigate the possible relationship between sociodemographic variables, depression, anxiety, stress, and burnout. Variables significant at *p* < 0.25 were subsequently entered into a stepwise multivariate logistic regression model. Reference categories for the categorical independent variables were chosen based on guidelines recommended by Johfre and Freese (38). For variables that categorize a quantity or rank (age, seniority, depression, anxiety, stress levels), the smallest quantities or lowest ranks are chosen as the reference groups (18–30 years old, entry level, normal or mild levels of depression, anxiety, and stress levels). For variables that unfold from a single group, such as relationship status, average hours worked per week, current work setup, and job satisfaction, the normative groups (single, 40–50 h per week, fully onsite, extremely satisfied) are chosen as the reference groups. For variables with symmetric categories (gender, country), groups that result in positive coefficient estimates are chosen as the reference groups.

Model fit was assessed using Hosmer & Lemeshow’s omnibus *χ*2 test, and we further report the final model’s McFadden’s adjusted R2, Nagelkerke’s R2, and Akaike information criterion (AIC). As all predictor variables in the model were categorical in nature, linearity assumptions were thus not applicable. Multicollinearity checks were conducted to ensure no multicollinearity between all predictors (GVIF<5.00). *A priori* sample size calculations following Bujang et al.’s (39) rule of thumb of *n* = 100 + 50i, where i refers to the number of independent variables in the final logistic regression model, revealed that a minimum sample size of *n* = 700 was sufficient to detect accurate estimates.

## 3 Results

### 3.1 Participant characteristics

**Figure 1:**
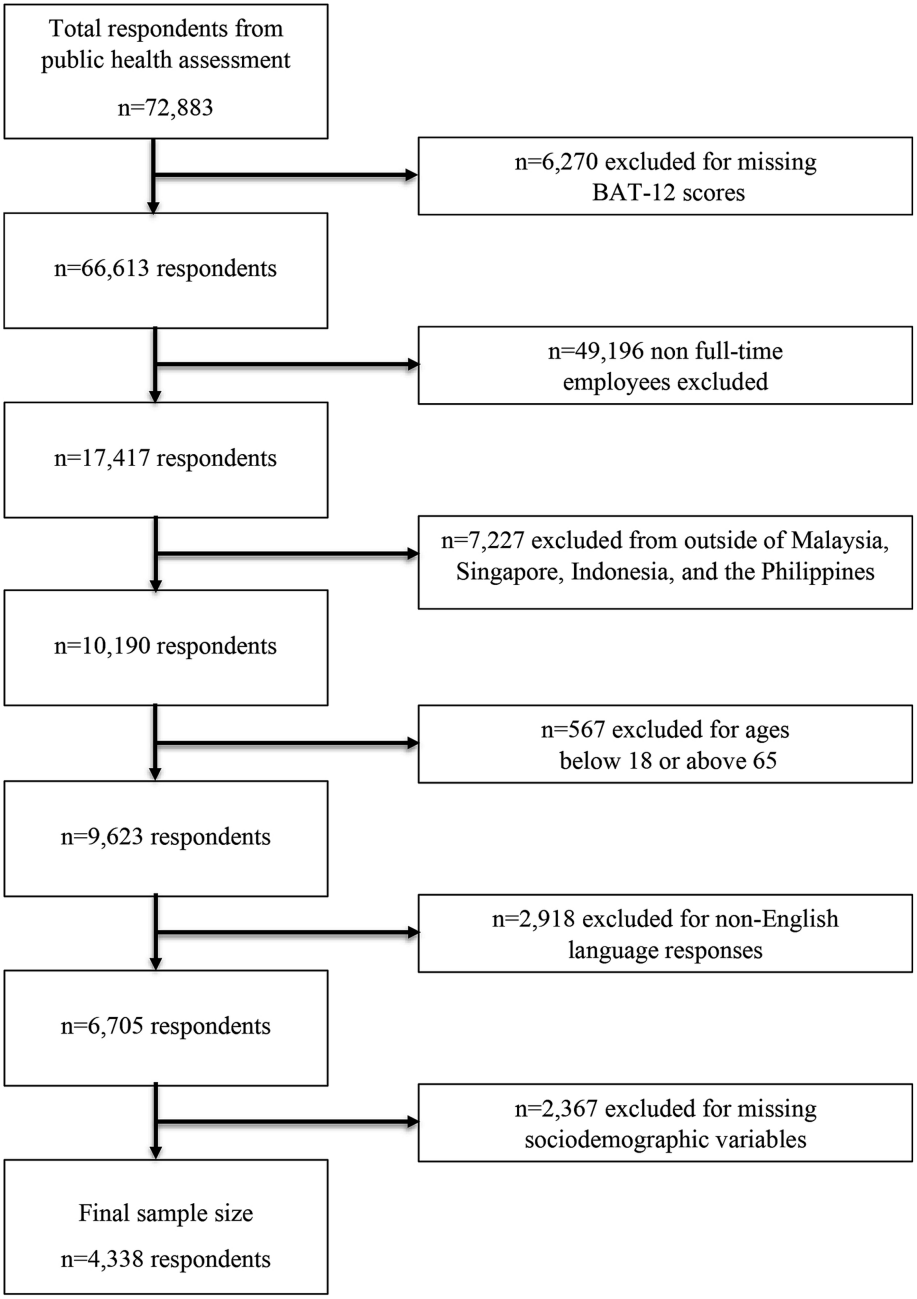
Flow chart showing participant inclusion flow into the study’s final sample size.

Out of the 72,883 responses in the public health assessment, 4,338 respondents fulfilled the study’s inclusion criteria, leaving a final response rate of 5.95%. Figure 1 demonstrates the flow of participant inclusion and exclusion based on the pre-set inclusion criteria. The median age of our sample was 29 (Interquartile range = 9.0). A majority of the sample were female (74.48%), aged 18–29 years old (53.69%), single (37.00%) and resided in Malaysia (54.89%). With regards to work demographic, our sample mostly worked in the education and training industry (8.41%), reported themselves as non-managerial executives (30.90%), worked 40–50 hours a week (47.10%), worked fully onsite (i.e., in-office) at the time (53.39%), and were moderately satisfied with their job (30.45%). Detailed sample characteristics are reported in Table 1.

**Table 1:** Sociodemographic characteristics of the sample (*N* = 4,338).

	N	%
Gender		
Male	1,092	25.17%
Female	3,231	74.48%
Other	15	0.35%
Age		
18–29	2,329	53.69%
30–39	1,448	33.38%
40–49	433	9.98%
50–65	128	2.95%
Country		
Malaysia	2,381	54.89%
Singapore	401	9.24%
Indonesia	337	7.77%
Philippines	1,219	28.10%
Relationship status		
Single	1,605	37.00%
Casually dating	361	8.32%
In a long-term relationship	773	17.82%
Married or in a domestic partnership	1,490	34.35%
Divorced, or separated	92	2.12%
Widowed	17	0.39%
Industry		
Science & Technology	59	1.36%
Education & Training	365	8.41%
Administration & Office Support	322	7.42%
Mining, Resources & Energy	53	1.22%
Manufacturing, Transport & Logistics	217	5.00%
Accounting	252	5.81%
Engineering	222	5.12%
Sales	98	2.26%
Call Center & Customer Service	341	7.86%
Banking & Financial Services	300	6.92%
Trades & Services	31	0.71%
Information & Communication Technology	257	5.92%
Healthcare & Medical	339	7.81%
Advertising, Arts & Media	123	2.84%
Retail & Consumer Products	139	3.20%
Hospitality & Tourism	94	2.17%
Construction	165	3.80%
Human Resources & Recruitment	133	3.07%
Design & Architecture	46	1.06%
Legal	60	1.38%
Consulting & Strategy	92	2.12%
Real Estate & Property	61	1.41%
Government & Defense	118	2.72%
Marketing & Communications	127	2.93%
Community Services & Development	36	0.83%
Sport and Recreation	9	0.21%
Insurance & Superannuation	52	1.12%
Farming, Animals & Conservation	16	0.37%
Others	211	4.86%
Seniority		
Senior management	265	4.61%
Middle management	893	15.52%
Lower management	1,201	20.87%
Non-managerial executive	1778	30.90%
Entry level	1,240	21.55%
Not applicable	377	6.55%
Average hours worked per week		
Less than 40 hours per week	2,451	38.75%
40–50 hours per week	2043	47.10%
More than 50 hours per week	614	14.15%
Current work setup		
Fully onsite	2,316	53.39%
Mostly onsite with some remote work	756	17.43%
Mostly remote with some onsite work	754	17.38%
Fully remote	512	11.80%
Work satisfaction		
Extremely satisfied	136	3.14%
Very satisfied	542	12.49%
Moderately satisfied	1,321	30.45%
Neither dissatisfied nor satisfied	912	21.02%
Moderately dissatisfied	767	17.68%
Very dissatisfied	397	9.15%
Extremely dissatisfied	263	6.06%

### 3.2 Burnout and psychological distress

The prevalence of burnout, depression, anxiety, and stress for each level of severity in each country are shown in Tables 2, 3. Across the four countries, a majority of respondents reported high (33.93%) or very high (28.98%) levels of burnout. Similar patterns are reported for respondents experiencing severe (10.88%) or extremely severe (37.37%) symptoms of anxiety, and severe (14.18%) or extremely severe (36.91%) depression. In comparison, the prevalence of severe or extremely severe symptoms of stress across our sample was only 20.40 and 15.81%, respectively.

**Table 2:** Prevalence of burnout in the region and across the four countries.

			Burnout
		*N*	% (95% CI)
Malaysia	Low	158	6.64 (5.64–7.64)
	Average	839	35.24 (33.31–37.16)
	High	778	32.68 (31.00–34.56)
	Very High	606	25.45 (23.70–27.20)
Singapore	Low	17	4.24 (2.27–6.21)
	Average	116	28.93 (24.49–33.37)
	High	164	40.90 (36.01–45.71)
	Very High	104	25.94 (21.65–30.22)
Indonesia	Low	16	4.74 (2.48–7.02)
	Average	106	31.45 (26.50–36.41)
	High	126	37.38 (32.22–42.55)
	Very High	89	26.40 (21.70–31.11)
Philippines	Low	48	3.94 (2.85–5.03)
	Average	309	25.35 (22.91–27.79)
	High	404	33.14 (30.50–35.78)
	Very High	458	37.57 (34.85–40.29)
Total	Low	239	5.51 (4.83–6.19)
	Average	1,370	31.58 (30.20–32.96)
	High	1,472	33.93 (32.52–35.34)
	Very High	1,257	28.98 (27.63–30.33)

**Table 3:** Prevalence of anxiety, depression, and stress in the region and across the four countries.

			Anxiety		Depression		Stress
		N	% (95% CI)	N	% (95% CI)	N	% (95% CI)
Malaysia	Normal	802	33.73 (31.83–35.62)	666	27.97 (26.17–29.77)	1,009	42.38 (40.39–44.36)
	Mild	174	7.31 (6.26–8.35)	230	9.66 (8.47–10.85)	264	11.09 (9.83–12.35)
	Moderate	440	18.48 (16.92–20.03)	453	19.03 (17.45–20.60)	364	15.29 (13.84–16.73)
	Severe	220	9.24 (8.08–10.40)	315	13.23 (11.87–14.59)	406	17.05 (15.54–18.56)
	Extremely Severe	774	32.51 (30.63–34.39)	717	30.11 (28.27–31.96)	338	14.20 (12.79–15.60)
Singapore	Normal	103	25.69 (21.41–29.96)	77	19.20 (15.35–23.06)	116	28.93 (24.49–33.36)
	Mild	27	6.73 (4.28–9.19)	28	6.98 (4.49–9.48)	54	13.47 (10.13–16.81)
	Moderate	89	22.19 (18.13–26.26)	80	19.95 (16.04–23.86)	94	23.44 (19.30–27.59)
	Severe	47	11.72 (8.57–14.87)	68	16.96 (13.28–20.63)	90	22.44 (18.36–26.53)
	Extremely Severe	135	33.67 (29.04–38.29)	148	36.91 (32.18–41.63)	47	11.72 (8.57–14.87)
Indonesia	Normal	64	18.99 (14.80–23.18)	63	18.69 (14.53–22.86)	89	26.41 (21.70–31.12)
	Mild	18	5.34 (2.94–7.74)	23	6.82 (4.13–9.52)	51	15.13 (11.31–18.96)
	Moderate	72	21.36 (16.99–25.74)	64	18.99 (14.80–23.18)	72	21.36 (16.99–25.74)
	Severe	56	16.62 (12.64–20.59)	57	16.91 (12.91–20.91)	67	19.88 (15.62–24.14)
	Extremely Severe	127	37.69 (32.51–42.86)	130	38.58 (33.38–43.77)	58	17.21 (13.18–21.24)
Philippines	Normal	203	16.65 (14.56–18.74)	163	13.37 (11.46–15.28)	284	23.30 (20.92–25.67)
	Mild	64	5.25 (4.00–6.50)	88	7.22 (5.77–8.67)	142	11.65 (9.85–13.45)
	Moderate	188	15.42 (13.40–17.45)	187	15.34 (13.32–17.36)	228	18.70 (16.51–20.89)
	Severe	149	12.22 (10.38–14.06)	175	14.36 (12.39–16.32)	322	26.42 (23.94–28.89)
	Extremely Severe	615	50.45 (47.64–53.26)	606	49.71 (46.91–52.52)	243	19.93 (17.69–22.18)
Total	Normal	1,173	27.04 (25.72–28.36)	969	22.34 (21.10–23.58)	1,498	34.53 (33.12–35.95)
	Mild	283	6.52 (5.79–7.26)	369	8.51 (7.68–9.34)	511	11.78 (10.82–12.74)
	Moderate	789	18.19 (17.04–19.34)	784	18.07 (16.93–19.22)	758	17.47 (16.34–18.60)
	Severe	472	10.88 (9.95–11.81)	615	14.18 (13.14–15.22)	885	20.40 (19.20–21.60)
	Extremely Severe	1,621	37.37 (35.93–38.81)	1,601	36.91 (35.47–38.34)	686	15.81 (14.73–16.90)

The prevalence of high or very high levels of burnout was the highest in the Philippines sample (70.71%), followed by Singapore (66.84%). Among the four countries, respondents from the Philippines also reported the highest prevalence of severe and above symptoms of anxiety (62.67%), depression (64.07%), and stress (46.55%). Respondents in Indonesia reported the second-highest prevalence for severe and above symptoms of anxiety (54.3%), depression (55.49%), and stress (39.09%). Meanwhile, respondents in Malaysia reported the lowest prevalence for severe and above symptoms of anxiety (41.75%), depression (43.34%), and stress (31.25%).

### 3.3 Factors associated with high or very high burnout

Univariate logistic regressions showed that sociodemographic variables, work characteristics, and psychological distress variables were all significantly associated with experiencing high to very high levels of work burnout (Supplementary Table S3). Table 4 presents the results of a multivariate logistic regression with the aforementioned variables as predictors of burnout.

**Table 4:** Association between sociodemographic variables and psychological distress with burnout.

	Burnout		
Variable	Odds Ratios (OR)	95% CIs	*p*-values
Gender			
Male	1.00		
Female	1.22	1.00–1.49	0.055
Other	1.42	0.32–7.32	0.658
Age			
18–29	1.00		
30–39	0.83	0.67–1.03	0.088
40–49	0.86	0.62–1.19	0.363
50–65	0.86	0.49–1.51	0.614
Country			
Malaysia	1.00		
Singapore	1.00	0.73–1.36	0.949
Indonesia	0.69	0.50–0.96	**0.026**
Philippines	1.10	0.87–1.38	0.421
Relationship status			
Single	1.00		
Casually dating	0.83	0.58–1.14	0.222
In a long-term relationship	0.77	0.60–1.00	0.050
Married or in a domestic partnership	0.98	0.78–1.22	0.827
Divorced, or separated	0.90	0.49–1.67	0.727
Widowed	0.52	0.12–2.20	0.382
Industry			
Science & Technology	1.00		
Education & Training	0.76	0.35–1.64	0.496
Administration & Office Support	0.82	0.37–1.78	0.621
Mining, Resources & Energy	0.79	0.27–2.29	0.673
Manufacturing, Transport & Logistics	0.73	0.32–1.64	0.456
Accounting	0.84	0.37–1.84	0.660
Engineering	0.90	0.40–2.01	0.806
Sales	0.96	0.38–2.43	0.934
Call Center & Customer Service	0.78	0.35–1.69	0.530
Banking & Financial Services	0.80	0.36–1.74	0.578
Trades & Services	1.57	0.44–5.97	0.498
Information & Communication Technology	0.75	0.34–1.65	0.482
Healthcare & Medical	0.80	0.36–1.73	0.578
Advertising, Arts & Media	0.50	0.21–1.18	0.116
Retail & Consumer Products	0.93	0.39–2.15	0.862
Hospitality & Tourism	1.49	0.57–3.87	0.417
Construction	0.84	0.36–1.92	0.676
Human Resources & Recruitment	1.35	0.56–3.22	0.506
Design & Architecture	0.64	0.22–1.87	0.417
Legal	0.87	0.31–2.41	0.787
Consulting & Strategy	1.11	0.45–2.68	0.819
Real Estate & Property	1.82	0.65–5.05	0.252
Government & Defense	0.95	0.38–2.34	0.904
Marketing & Communications	1.11	0.45–2.68	0.819
Community Services & Development	0.66	0.21–2.09	0.488
Sport and Recreation	0.85	0.07–5.78	0.886
Insurance & Superannuation	0.44	0.15–1.26	0.130
Farming, Animals & Conservation	1.30	0.24–8.83	0.775
Others	1.19	0.52–2.68	0.673
Seniority			
Entry level	1.00		
Senior management	0.85	0.53–1.37	0.509
Middle management	1.19	0.86–1.64	0.294
Lower management	1.18	0.89–1.56	0.259
Non-managerial executive	1.11	0.85–1.43	0.444
Not applicable	1.14	0.73–1.79	0.561
Average hours worked per week			
40–50 hours per week	1.00		
Less than 40 hours per week	1.23	1.02–1.48	**0.034**
More than 50 hours per week	1.36	1.03–1.81	**0.030**
Current work setup			
Fully onsite	1.00		
Mostly onsite with some remote work	1.08	0.85–1.37	0.542
Mostly remote with some onsite work	0.96	0.75–1.22	0.718
Fully remote	0.85	0.64–1.15	0.296
Work satisfaction			
Extremely satisfied	1.00		
Very satisfied	1.04	0.60–1.80	0.889
Moderately satisfied	3.04	1.82–5.10	**<0.001**
Neither dissatisfied nor satisfied	5.12	3.03–8.72	**<0.001**
Moderately dissatisfied	7.68	4.50–13.23	**<0.001**
Very dissatisfied	16.18	8.82–30.05	**<0.001**
Extremely dissatisfied	8.79	4.51–17.47	**<0.001**
Depression			
Normal or mild	1.00		
Moderate	3.04	2.41–3.83	**<0.001**
Severe or extremely severe	6.39	4.98–8.21	**<0.001**
Anxiety			
Normal or mild	1.00		
Moderate	1.99	1.58–2.52	**<0.001**
Severe or extremely severe	2.25	1.75–2.88	**<0.001**
Stress			
Normal or mild	1.00		
Moderate	2.17	1.70–2.78	**<0.001**
Severe or extremely severe	5.50	4.11–7.39	**<0.001**

Bolded *p*-values represent *p* < 0.005. McFadden’s adjusted *R*^2^ = 0.598; Cragg-Uhler (Nagelkerke) *R*^2^ = 0.755; Akaike information criterion (AIC) = 3513.820; Hosmer & Lemeshow test *χ*2 = 5.884, *p* > 0.05; Multicollinearity checks indicated no multicollinearity between all listed factors (GVIF < 5.00).

Compared to Malaysia, employees in Indonesia (AOR = 0.69, *p* < 0.05) had significantly lower odds of experiencing burnout. Separately, employees who worked either less than 40 hours per week (AOR = 1.23, *p* < 0.05) or more than 50 hours per week (AOR = 1.36, *p* < 0.05) reported significantly higher odds of experiencing burnout compared to employees who maintained the regular average of 40–50 regular work hours per week. Increasing job dissatisfaction was linked to higher risks of experiencing burnout, with employees who are very dissatisfied having the highest odds of experiencing burnout compared to those who are extremely satisfied (AOR = 16.46, *p* < 0.001). With regards to psychological distress, compared to those reporting normal or mild symptoms, employees in the region who reported moderate or above symptoms of depression, anxiety, and stress all reported higher odds of experiencing burnout (*p* < 0.001). Despite having significant results at a univariate level, no significant associations were detected between burnout and gender, relationship status, employment industry, work seniority, and current work arrangement (i.e., in-office, remote, hybrid). Country-level analyses investigating associated factors of burnout within each country are presented in Supplementary Tables S4–S7.

## 4 Discussion

Using retrospective data obtained from a large-scale public mental health assessment, we investigated the prevalence of burnout and its associated factors among the general working population of full-time employees in four countries in Southeast Asia. Across the four countries, 62.91% of respondents reported experiencing high or very high levels of burnout. Inter-country variations revealed that the prevalence of burnout was highest in the Philippines (70.71%) and Singapore (66.84%), and lowest in Malaysia (58.13%). As a secondary objective, we also found that 51.09% of respondents in the region were reporting severe and above symptoms of depression, followed by a 48.25% prevalence of anxiety, and a 36.21% prevalence of stress. The magnitude of burnout and psychological distress identified in this study highlights the rising necessity to pay attention to employee mental health and well-being in the region.

Limited evidence exists on the prevalence of burnout in the general working population and across occupational industries (40). To our knowledge, this study is the first in the region to investigate the prevalence of employee burnout in the general working adult population of Southeast Asia. Ndongo et al. recorded a 67.9% prevalence of burnout across industry sectors in Cameroon (41). Closer to the region, Matsuo et al. found that 31.0% of the general working population of Japan was experiencing burnout (40), while Lam et al. observed that 60% of corporate employees in Hong Kong were reporting moderate to high levels of emotional exhaustion, one of the traditionally measured components of burnout (42). The usage of different measures to assess burnout prevalence limits a straightforward comparison of the findings, though the prevalence of burnout we recorded in all four respective countries is highly similar to those reported by Ndongo et al. and Lam et al. (41, 42). Otherwise, Teo et al. reported a 20.0% prevalence of burnout among healthcare workers in Southeast Asia, with those in Singapore reporting the highest prevalence of 39.0% (43). However, it is difficult to speculate on the mechanisms behind the reported differences given that Teo et al.’s study focused on an entirely different, more specific employee population than ours.

In terms of work-related risk factors, we found that both working more and less than 40–50 h a week – the average weekly mandated work hours in the region – were associated with higher odds of burnout in employees. Employees in Asia are typically more prone to working long and inflexible work hours in the face of rising work demands, largely owing to a strong cultural emphasis on work as a means of fulfilling social and familial responsibilities, and high levels of power distance that inhibit employees from voicing discontent over or refusing increasing workloads (44). Our findings are consistent with previous studies in the region linking more than usual work hours and higher burnout risk (40, 43). Surprisingly, we also found that employees who worked less than the average mandated weekly hours were also at higher risk of burnout, though the odds are slightly lesser compared to those working more than 50 hours a week. While shorter working hours have been generally linked to improved work quality and work-life balance (45, 46), existing research does indicate that the relationship between reduced work hours and employee health and well-being can be unclear (46), warranting a need for future studies in this area to investigate the role of potential moderators (47–49). Additionally, cultural attitudes may contribute to differences in how Asians view working hours, as cultural values such as social harmony, collectivism, and respect for authority may translate to a higher appreciation for longer working hours (50). If anything, our results indicate that employees in the region may require participating in a minimum number of working hours per week to consider themselves productive and equal contributors in the workplace, the absence of which may negatively impact employees’ self-efficacy, which under the Social Cognitive Theory can make them more prone to developing burnout (10, 50, 51).

Furthermore, our results revealed that job satisfaction was significantly associated with burnout, with employees who are more dissatisfied with their work having higher odds of experiencing burnout. Previous work has established the negative relationship between job satisfaction and burnout (52–55), and how this relationship can lead to increased turnover intentions among employees (52, 56, 57). However, many employees in the region value job security, especially during uncertain economic conditions, and are thus less likely to act on their work dissatisfaction compared to their Western counterparts (58). Nevertheless, employees in Southeast Asia are traditionally faced with high work demands, work overload, work-life imbalance (44), and wage stagnation (59, 60), all of which largely contribute to reduced job satisfaction (61–63). Given the adverse organizational consequences that burnout can bring, our findings highlight the importance of addressing work dissatisfaction as part of burnout prevention among employees.

Despite the large number of studies dedicated to understanding the relationship between remote work and employee wellbeing since the emergence of COVID-19, we found no significant association between different kinds of work arrangements and burnout among the employees in the region. The existing literature in the area of remote work has so far been conflicting. Although multiple studies have established the benefits of remote work arrangements and its impact in reducing work–family conflict, improving work-life balance, work efficiency, and employee mental health (64–67), there is also an equivalent amount of evidence to suggest a negative relationship between remote or hybrid work arrangements and employee wellbeing, with remote employees being more vulnerable to increased burnout, escalating job demands, poorer self-rated mental health, intensified physical and mental exhaustion, and increased presenteeism (65, 67–70). Thus, our results further support the suggestion that an indirect relationship likely exists between remote work and employee well-being. As employees continue to demand remote and flexible work arrangements post-pandemic, there is a need for more studies in the area to establish the moderators of this relationship among employees in the region to ensure that organizations are well-equipped to manage the risks that come with remote work arrangements.

Our results reveal no significant relationship between gender and burnout, further adding to the inconsistent literature that exists in the area. Purvanova and Muros’ meta-analysis of gender differences in burnout found that, while women tend to score higher on burnout measures than men, women are significantly likelier to report experiencing emotional exhaustion, whereas men are more likely to report experiencing the depersonalisation component of burnout (71). Additionally, despite a population-based study in Sweden showing that more women than men suffer from burnout, this difference was only a function of age (11), and became non-existent once all other factors were taken into account (12). Separately, when marital status is taken into account, single men and married women tend to be at higher risk of burnout compared to their counterparts (10, 13), though this association has been inconclusive in the literature (13). Our findings thus contribute to the growing body of evidence suggesting that gender alone cannot explain the difference in reports of burnout between the different gender groups (72), thus highlighting the need for more studies in the region to look into potential moderators to further understand the nuance in the relationship between gender and burnout.

While previous studies have linked the rise in COVID-19 cases and social restrictions as a contributor to deteriorating mental health (73, 74), our findings indicate a long-lasting psychological impact of the pandemic, as we continue to observe an overall decline in mental well-being in the region despite lessening COVID-19 cases and the removal of most pandemic social restrictions in 2022 (75, 76). We recorded a higher prevalence of depression, anxiety and stress symptoms than those reported in Tay et al.’s study, which reported a regional prevalence of 48.86% for depression, 49.34% for anxiety, and 36.19% for stress in the general population in 2021 (77). At the time of our data collection (October 2022), most of the countries in Southeast Asia were only beginning to undergo economic recovery post-pandemic (78), which meant that employees in the region were facing high economic pressures - not only to recover from the economic and financial impact of the pandemic (73, 79), but also to face global inflation and the rising cost of living at the time (78). In addition, as we found that experiencing moderate and above symptoms of depression, anxiety, and stress significantly increased the odds of employee burnout, it is also possible that the high prevalence rates we recorded here reflect the long-term patterns of rising mental health challenges throughout the region (80, 81), which argues for the importance of effective intervention and early prevention efforts to mitigate the deterioration of mental well-being in the region.

Several limitations should be acknowledged in assessing this study’s findings. Firstly, this study utilized the BAT-12 to measure burnout due to the scale’s ability to reliably provide an overall score of burnout, as well as its validated scoring classification (6), both of which were integral to the objectives of the study. However, the usage of BAT-12 over more traditional burnout measures such as the Maslach Burnout Inventory or the Copenhagen Burnout Inventory limits the direct comparisons of our findings against existing research in this field. Secondly, we did not include more elaborate work-related factors such as emotional labor, job autonomy, inter-role conflict, and social support (10), which could have provided more insight into documenting the burnout phenomenon in the region. Additionally, the nature and source of data used in this study may be a possible source of bias, as individuals who were attracted and opted to complete the online mental health assessment were more than likely to come from those with a higher degree of awareness of the importance of mental wellbeing. In turn, this may have resulted in prevalence estimates that are not reflective of a purely random and mixed sample. Separately, the logistic regression results for job satisfaction reveal wide confidence intervals for the adjusted odd ratios as dissatisfaction increases, suggesting less precise estimates that warrant further caution in interpreting the large odd ratios. In addition, our sample consists of a higher proportion of residents from Malaysia (54.896%) and the Philippines (28.10%), which limits the representability of our findings across countries. Separately, we elected to exclude participants with missing data which may have introduced selection bias in our study’s population (82).

Furthermore, several of the study’s limitations can be attributed to the cross-sectional design of the study. Firstly, the objectives of the study are to investigate the associated factors that contribute to the development of burnout among employees in the region. However, as a cross-sectional study, no causal relationships can be inferred from the results of our study. Secondly, as a cross-sectional study that utilizes self-report measures, the results of this study are susceptible to common-method bias which can compromise the construct validity and reliability, and inflate the relationships between our observed variables (83). Finally, as a cross-sectional study, our results were only able to capture the mental health status of employees in the region as of October 2022. Given the rapid and mass social, political and economic changes afflicting the region these past few years, and the dynamic nature of burnout itself (84), our findings hold limited temporal generalisability, warranting the need for more studies in the future that look into employee burnout and mental health in the Southeast Asian region.

In conclusion, this study looked into the prevalence of burnout among the general working population of Southeast Asia and provides evidence of rising mental health concerns across employees in the region. We found that the prevalence of burnout in the region was generally high, and that a majority of the employees are also dealing with heightened symptoms of psychological distress such as depression, anxiety, and stress. Working longer and shorter hours than the weekly average, having lower job satisfaction and having symptoms of anxiety, depression, and stress were associated with higher odds of experiencing burnout. Even as the region moves toward a post-pandemic landscape, employees are still dealing with the long-term economic and psychological impact of the pandemic, and our findings crucially highlight the importance of burnout prevention and intervention in the region.

## Data availability statement

The raw data supporting the conclusions of this article will be made available by the authors, without undue reservation.

## Ethics statement

The studies involving humans were approved by Medical Research & Ethics Committee, Ministry of Health Malaysia. The studies were conducted in accordance with the local legislation and institutional requirements. The ethics committee/institutional review board waived the requirement of written informed consent for participation from the participants or the participants’ legal guardians/next of kin because the data used was retrospective secondary data obtained from a deidentified database.

## Author contributions

AFAA: Conceptualization, Formal analysis, Methodology, Project administration, Writing – original draft, Writing – review & editing. TO: Conceptualization, Supervision, Writing – review & editing.
