# Supplementary material for: Prevalence and associated factors of burnout among working adults in Southeast Asia: results from a public health assessment
Source: Front Public Health. 2024 Mar 14;12:1326227. doi: 10.3389/fpubh.2024.1326227 (PMC10972957; doi:10.3389/fpubh.2024.1326227)
Supplement: Supplementary file 1 [file Table_1.DOCX]

Supplementary Material

**Prevalence and Associated Factors of Burnout Among Working Adults in Southeast Asia: Results from a Public Health Assessment**

**Amani Fadzlina Abdul Aziz^1^, Tiffanie Ong^1*^**

*** Correspondence:** Tiffanie Ong: tiffanie@naluri.life

# Supplementary Tables

**Table 1**. STROBE Statement—Checklist of items that should be included in reports of cross-sectional studies

|  | Item No | Recommendation | Page No |
| --- | --- | --- | --- |
| **Title and abstract** | 1 | (*a*) Indicate the study’s design with a commonly used term in the title or the abstract | 1 |
|  |  | (*b*) Provide in the abstract an informative and balanced summary of what was done and what was found | 1 |
| Introduction | | | |
| Background/rationale | 2 | Explain the scientific background and rationale for the investigation being reported | 3-4 |
| Objectives | 3 | State specific objectives, including any prespecified hypotheses | 3-4 |
| Methods | | | |
| Study design | 4 | Present key elements of study design early in the paper | 4 |
| Setting | 5 | Describe the setting, locations, and relevant dates, including periods of recruitment, exposure, follow-up, and data collection | 4 |
| Participants | 6 | (*a*) Give the eligibility criteria, and the sources and methods of selection of participants | 5 |
| Variables | 7 | Clearly define all outcomes, exposures, predictors, potential confounders, and effect modifiers. Give diagnostic criteria, if applicable | 6-7 |
| Data sources/ measurement | 8* | For each variable of interest, give sources of data and details of methods of assessment (measurement). Describe comparability of assessment methods if there is more than one group | 6-7 |
| Bias | 9 | Describe any efforts to address potential sources of bias | 5-6 |
| Study size | 10 | Explain how the study size was arrived at | 5 |
| Quantitative variables | 11 | Explain how quantitative variables were handled in the analyses. If applicable, describe which groupings were chosen and why | 6-7 |
| Statistical methods | 12 | (*a*) Describe all statistical methods, including those used to control for confounding | 8 |
|  |  | (*b*) Describe any methods used to examine subgroups and interactions | 8 |
|  |  | (*c*) Explain how missing data were addressed | 5 |
|  |  | (*d*) If applicable, describe analytical methods taking account of sampling strategy | NA |
|  |  | (*e*) Describe any sensitivity analyses | NA |
| Results | | | |
| Participants | 13* | (a) Report numbers of individuals at each stage of study—eg numbers potentially eligible, examined for eligibility, confirmed eligible, included in the study, completing follow-up, and analysed | 8 |
|  |  | (b) Give reasons for non-participation at each stage | NA |
|  |  | (c) Consider use of a flow diagram | NA |
| Descriptive data | 14* | (a) Give characteristics of study participants (eg demographic, clinical, social) and information on exposures and potential confounders | 8-9 |
|  |  | (b) Indicate number of participants with missing data for each variable of interest | NA |
| Outcome data | 15* | Report numbers of outcome events or summary measures | 9 |
| Main results | 16 | (*a*) Give unadjusted estimates and, if applicable, confounder-adjusted estimates and their precision (eg, 95% confidence interval). Make clear which confounders were adjusted for and why they were included | 9-10 |
|  |  | (*b*) Report category boundaries when continuous variables were categorized | 9-10 |
|  |  | (*c*) If relevant, consider translating estimates of relative risk into absolute risk for a meaningful time period | N/A |
| Other analyses | 17 | Report other analyses done—eg analyses of subgroups and interactions, and sensitivity analyses | N/A |
| Discussion | | | |
| Key results | 18 | Summarise key results with reference to study objectives | 10-11 |
| Limitations | 19 | Discuss limitations of the study, taking into account sources of potential bias or imprecision. Discuss both direction and magnitude of any potential bias | 14-15 |
| Interpretation | 20 | Give a cautious overall interpretation of results considering objectives, limitations, multiplicity of analyses, results from similar studies, and other relevant evidence | 11-14 |
| Generalisability | 21 | Discuss the generalisability (external validity) of the study results | 15 |
| Other information | | | |
| Funding | 22 | Give the source of funding and the role of the funders for the present study and, if applicable, for the original study on which the present article is based | Title Page |

*Give information separately for exposed and unexposed groups.

**Table 2.** Means and standard deviations for burnout, depression, anxiety, and stress for the overall sample and by sociodemographic group.

|  |  |  | **Burnout** | **Depression** | **Anxiety** | **Stress** |
| --- | --- | --- | --- | --- | --- | --- |
|  | N | % | M (SD) | M (SD) | M (SD) | M (SD) |
| **Total** |  |  |  |  |  |  |
| Total sample | 4338 | 100.00% | 3.12 (0.95) | 21.25 (12.76) | 15.75 (11.03) | 20.62 (10.96) |
| **Gender** |  |  |  |  |  |  |
| Male | 1092 | 25.17 % | 2.90 (0.96) | 18.84 (12.79) | 12.94 (10.30) | 17.76 (10.82) |
| Female | 3231 | 74.48 % | 3.19 (0.94) | 22.06 (12.65) | 16.69 (11.11) | 21.58 (10.84) |
| Other | 15 | 0.35 % | 3.36 (0.98) | 23.87 (11.96) | 20.13 (12.65) | 21.87 (10.73) |
| **Age** |  |  |  |  |  |  |
| 18-29 | 2329 | 53.69 % | 3.29 (0.92) | 24.06 (12.09) | 18.17 (10.92) | 22.69 (10.42) |
| 30-39 | 1448 | 33.38 % | 3.02 (0.94) | 19.74 (12.73) | 14.06 (10.62) | 19.62 (11.07) |
| 40-49 | 433 | 9.98 % | 2.73 (0.89) | 14.76 (11.70) | 11.01 (9.73) | 15.69(10.24) |
| 50+ | 128 | 2.95 % | 2.29 (0.85) | 9.31 (10.50) | 6.92 (8.23) | 10.89 (9.39) |
| **Country** |  |  |  |  |  |  |
| Malaysia | 2381 | 54.89 % | 3.07 (0.96) | 20.06 (12.75) | 15.10 (11.18) | 19.88 (11.37) |
| Singapore | 401 | 9.24 % | 3.20 (0.88) | 22.95 (12.47) | 15.61 (10.39) | 21.59 (10.34) |
| Indonesia | 337 | 7.77 % | 3.19 (0.93) | 23.61 (12.02) | 18.55 (10.43) | 23.16 (10.30) |
| Philippines | 1219 | 28.10 % | 3.36 (0.96) | 25.44 (12.19) | 19.89 (11.13) | 23.79 (10.43) |
| **Relationship status** |  |  |  |  |  |  |
| Single | 1605 | 37.00 % | 3.22 (0.94) | 23.08 (12.61) | 16.36 (10.98) | 20.99 (10.71) |
| Casually dating | 361 | 8.32 % | 3.25 (0.92) | 24.04 (11.96) | 18.38 (11.26) | 23.00 (10.47) |
| In a long-term relationship | 773 | 17.82 % | 3.28 (0.93) | 24.12 (12.25) | 18.55 (11.18) | 23.59 (10.62) |
| Married or in a domestic partnership | 1490 | 34.35 % | 2.89 (0.94) | 17.14 (12.31) | 12.96 (10.29) | 18.06 (10.88) |
| Divorced, or separated | 92 | 2.12 % | 3.17 (0.95) | 22.22 (12.80) | 16.78 (11.32) | 21.85 (11.72) |
| Widowed | 17 | 0.39 % | 2.53 (0.98) | 14.94 (13.97) | 14.35 (10.38) | 16.71 (11.79) |
| **Industry** |  |  |  |  |  |  |
| Science & Technology | 59 | 1.36 % | 3.06 (0.75) | 21.52 (10.51) | 15.25 (10.33) | 19.29 (9.37) |
| Education & Training | 365 | 8.41 % | 3.02 (0.96) | 20.14 (12.78) | 14.39 (10.70) | 19.76 (10.54) |
| Administration & Office Support | 322 | 7.42 % | 3.18 (1.02) | 22.80 (12.84) | 17.49 (11.88) | 21.25 (11.09) |
| Mining, Resources & Energy | 53 | 1.22 % | 2.94 (0.99) | 17.55 (13.33) | 12.08 (10.27) | 17.47 (11.63) |
| Manufacturing, Transport & Logistics | 217 | 5.00 % | 3.13 (0.98) | 21.21 (12.60) | 16.26 (11.39) | 20.26 (11.02) |
| Accounting | 252 | 5.81 % | 3.15 (0.97) | 21.59 (12.49) | 16.21 (10.93) | 20.83 (11.04) |
| Engineering | 222 | 5.12 % | 3.08 (0.93) | 20.42 (12.22) | 14.99 (10.77) | 20.04 (10.70) |
| Sales | 98 | 2.26 % | 3.33 (0.94) | 22.53 (12.52) | 17.37 (10.95) | 22.73 (10.63) |
| Call Centre & Customer Service | 341 | 7.86 % | 3.40 (0.95) | 26.27 (12.14) | 20.53 (11.15) | 24.66 (10.64) |
| Banking & Financial Services | 300 | 6.92 % | 3.01 (0.98) | 19.37 (13.41) | 14.51 (11.03) | 19.63 (11.39) |
| Trades & Services | 31 | 0.71 % | 3.29 (0.72) | 22.19 (12.39) | 15.10 (10.92) | 21.16 (10.52) |
| Information & Communication Technology | 257 | 5.92 % | 3.09 (0.95) | 21.41 (12.79) | 16.38 (11.35) | 20.62 (11.18) |
| Healthcare & Medical | 339 | 7.81 % | 2.99 (0.96) | 19.85 (12.78) | 14.15 (10.75) | 19.72 (11.08) |
| Advertising, Arts & Media | 123 | 2.84 % | 3.05 (0.79) | 21.41 (11.69) | 15.40 (9.64) | 21.54 (10.25) |
| Retail & Consumer Products | 139 | 3.20 % | 3.21 (0.88) | 22.17 (11.46) | 15.37 (9.95) | 20.52 (10.28) |
| Hospitality & Tourism | 94 | 2.17 % | 3.31 (0.85) | 25.32 (12.05) | 19.55 (10.64) | 23.83 (10.22) |
| Construction | 165 | 3.80 % | 2.93 (0.99) | 18.46 (13.40) | 13.02 (11.21) | 18.01 (11.37) |
| Human Resources & Recruitment | 133 | 3.07 % | 3.24 (0.86) | 21.41 (12.38) | 16.05 (10.41) | 21.29 (10.24) |
| Design & Architecture | 46 | 1.06 % | 3.05 (0.95) | 20.39 (13.34) | 15.48 (10.88) | 21.09 (11.36) |
| Legal | 60 | 1.38 % | 3.25 (0.93) | 22.53 (13.24) | 15.93 (10.47) | 20.43 (11.39) |
| Consulting & Strategy | 92 | 2.12 % | 2.97 (0.95) | 16.91 (11.50) | 12.65 (9.14) | 18.91 (10.03) |
| Real Estate & Property | 61 | 1.41 % | 3.13 (1.01) | 20.49 (12.75) | 13.87 (11.68) | 18.52 (10.51) |
| Government & Defence | 118 | 2.72 % | 3.08 (0.98) | 21.90 (12.84) | 15.78 (11.46) | 20.71 (11.42) |
| Marketing & Communications | 127 | 2.93 % | 3.10 (0.82) | 20.24 (12.37) | 15.17 (10.43) | 20.63 (10.38) |
| Community Services & Development | 36 | 0.83 % | 2.96 (0.97) | 18.83 (11.91) | 13.22 (9.48) | 17.94 (10.47) |
| Sport and Recreation | 9 | 0.21 % | 2.78 (1.08) | 14.67 (13.19) | 9.33 (9.11) | 14.67 (12.41) |
| Insurance & Superannuation | 52 | 1.12 % | 2.92 (0.87) | 17.27 (13.44) | 13.81 (11.11) | 18.04 (11.67) |
| Farming, Animals & Conservation | 16 | 0.37 % | 3.47 (0.83) | 25.38 (11.40) | 18.38 (9.07) | 22.25 (8.16) |
| Others | 211 | 4.86 % | 3.12 (0.97) | 21.24 (13.43) | 15.45 (10.83) | 20.45 (11.31) |
| **Seniority** |  |  |  |  |  |  |
| Senior management | 265 | 4.61 % | 2.83 (0.90) | 17.49 (13.04) | 13.52 (10.99) | 18.74 (11.45) |
| Middle management | 893 | 15.52 % | 2.89 (0.94) | 17.16 (12.51) | 12.59 (10.68) | 17.71 (10.86) |
| Lower management | 1201 | 20.87 % | 3.17 (0.99) | 20.37 (12.95) | 15.51 (11.07) | 20.12 (11.25) |
| Non-managerial executive | 1778 | 30.90 % | 3.08 (0.93) | 21.86 (12.51) | 15.51 (10.87) | 21.94 (10.84) |
| Entry level | 1240 | 21.55 % | 3.29 (0.90) | 24.97 (11.77) | 15.71 (10.69) | 23.17 (10.13) |
| Not applicable | 377 | 6.55 % | 3.18 (0.97) | 22.65 (13.00) | 16.50 (11.01) | 21.35 (10.98) |
| **Average hours worked per week** |  |  |  |  |  |  |
| Less than 40 hours per week | 2451 | 38.75 % | 3.05 (0.93) | 20.45 (12.81) | 14.67 (10.78) | 19.70 (10.83) |
| 40-50 hours per week | 2043 | 47.10% | 3.13 (0.98) | 21.77 (12.77) | 16.73 (11398) | 21.16 (11.21) |
| More than 50 hours per week | 614 | 14.15% | 3.29 (0.91) | 22.52 (12.40) | 16.68 (10.55) | 22.34 (10.40) |
| **Current work setup** |  |  |  |  |  |  |
| Fully onsite | 2316 | 53.39 % | 3.15 (0.96) | 21.76 (12.82) | 16.15 (11.17) | 20.76 (11.02) |
| Mostly onsite with some remote work | 756 | 17.43 % | 2.99 (0.92) | 19.02 (12.78) | 13.60 (10.58) | 19.16 (10.99) |
| Mostly remote with some onsite work | 754 | 17.38 % | 3.07 (0.91) | 20.69 (12.27) | 15.37 (10.46) | 20.53 (10.39) |
| Fully remote | 512 | 11.80 % | 3.21 (0.98) | 23.09 (12.60) | 17.70 (11.40) | 22.53 (11.20) |
| **Work satisfaction** |  |  |  |  |  |  |
| Extremely satisfied | 136 | 3.14 % | 2.38 (1.06) | 16.10 (14.16) | 13.25 (12.09) | 17.18 (12.79) |
| Very satisfied | 542 | 12.49 % | 2.42 (0.90) | 15.07 (12.84) | 12.03 (10.75) | 15.97 (10.95) |
| Moderately satisfied | 1321 | 30.45 % | 2.88 (0.84) | 17.92 (12.04) | 14.04 (10.51) | 18.50 (10.34) |
| Neither dissatisfied nor satisfied | 912 | 21.02 % | 3.30 (0.82) | 23.71 (11.70) | 16.98 (10.75) | 21.96 (10.24) |
| Moderately dissatisfied | 767 | 17.68 % | 3.35 (0.79) | 23.23 (11.45) | 16.70 (10.33) | 22.05 (10.08) |
| Very dissatisfied | 397 | 9.15 % | 3.65 (0.86) | 26.30 (11.81) | 19.28 (10.91) | 24.16 (10.64) |
| Extremely dissatisfied | 263 | 6.06 % | 4.00 (0.95) | 31.53 (11.18) | 23.52 (11.87) | 28.38 (10.92) |

**Table 3.** Crude odds ratios and 95% confidence intervals from univariate logistic regression.

|  | **Burnout** | | |
| --- | --- | --- | --- |
| Variable | Crude Odds Ratios | 95% CIs | p-values |
| **Gender** |  |  |  |
| Male | *Reference* |  |  |
| Female | 1.66 | 1.44 – 1.91 | **<0.001** |
| Other | 2.36 | 0.80 – 8.55 | 0.144 |
| **Age** |  |  |  |
| 18-29 | *Reference* |  |  |
| 30-39 | 0.59 | 0.51 – 0.68 | **<0.001** |
| 40-49 | 0.39 | 0.32 – 0.48 | **<0.001** |
| 50-65 | 0.17 | 0.11 – 0.25 | **<0.001** |
| **Country** |  |  |  |
| Malaysia | *Reference* |  |  |
| Singapore | 1.45 | 1.16 – 1.82 | **0.001** |
| Indonesia | 1.27 | 1.00 – 1.61 | **0.048** |
| Philippines | 1.74 | 1.50 – 2.02 | **<0.001** |
| **Relationship status** |  |  |  |
| Single | *Reference* |  |  |
| Casually dating | 1.06 | 0.83 – 1.36 | 0.630 |
| In a long-term relationship | 1.04 | 0.87 – 1.25 | 0.659 |
| Married or in a domestic partnership | 0.55 | 0.48 – 0.64 | **<0.001** |
| Divorced, or separated | 0.85 | 0.55 – 1.34 | **0.482** |
| Widowed | 0.26 | 0.09 – 0.69 | **0.008** |
| **Industry** |  |  |  |
| Science & Technology | *Reference* |  |  |
| Education & Training | 0.74 | 0.41 – 1.30 | 0.303 |
| Administration & Office Support | 0.99 | 0.55 – 1.76 | 0.986 |
| Mining, Resources & Energy | 0.57 | 0.27 – 1.22 | 0.151 |
| Manufacturing, Transport & Logistics | 1.00 | 0.54 – 1.82 | 0.988 |
| Accounting | 1.09 | 0.59 – 1.95 | 0.786 |
| Engineering | 0.94 | 0.51 – 1.70 | 0.849 |
| Sales | 1.25 | 0.63 – 2.48 | 0.519 |
| Call Centre & Customer Service | 1.50 | 0.82 – 2.66 | 0.177 |
| Banking & Financial Services | 0.75 | 0.42 – 1.33 | 0.337 |
| Trades & Services | 1.89 | 0.72 – 5.42 | 0.209 |
| Information & Communication Technology | 0.77 | 0.42 – 1.38 | 0.395 |
| Healthcare & Medical | 0.80 | 0.44 – 1.40 | 0.435 |
| Advertising, Arts & Media | 0.78 | 0.41 – 1.47 | 0.449 |
| Retail & Consumer Products | 1.12 | 0.58 – 2.11 | 0.734 |
| Hospitality & Tourism | 1.92 | 0.93 – 3.97 | 0.076 |
| Construction | 0.68 | 0.36 – 1.25 | 0.218 |
| Human Resources & Recruitment | 1.28 | 0.67 – 2.45 | 0.449 |
| Design & Architecture | 0.72 | 0.32 – 1.58 | 0.412 |
| Legal | 1.11 | 0.52 – 2.36 | 0.795 |
| Consulting & Strategy | 0.75 | 0.38 – 1.47 | 0.405 |
| Real Estate & Property | 1.05 | 0.50 – 2.24 | 0.893 |
| Government & Defence | 1.08 | 0.55 – 2.07 | 0.823 |
| Marketing & Communications | 1.04 | 0.54 – 1.98 | 0.900 |
| Community Services & Development | 0.62 | 0.26 – 1.44 | 0.263 |
| Sport and Recreation | 0.28 | 0.05 – 1.16 | 0.090 |
| Insurance & Superannuation | 0.47 | 0.22 – 1.01 | 0.055 |
| Farming, Animals & Conservation | 2.39 | 0.68 – 11.30 | 0.209 |
| Others | 1.00 | 0.54 – 1.82 | 0.995 |
| **Seniority** |  |  |  |
| Entry level | *Reference* |  |  |
| Senior management | 0.44 | 0.32 – 0.61 | **<0.001** |
| Middle management | 0.47 | 0.39 – 0.58 | **<0.001** |
| Lower management | 0.68 | 0.56 – 0.83 | **<0.001** |
| Non-managerial executive | 0.75 | 0.62 – 0.90 | **0.002** |
| Not applicable | 0.77 | 0.56 – 1.05 | **0.098** |
| **Average hours worked per week** |  |  |  |
| 40-50 hours a week | *Reference* |  |  |
| Less than 40 hours a week | 0.94 | 0.82 – 1.07 | 0.338 |
| More than 50 hours a week | 1.42 | 1.17 – 1.74 | **0.001** |
| **Current work setup** |  |  |  |
| Fully onsite | *Reference* |  |  |
| Mostly onsite with some remote work | 0.74 | 0.63 – 0.88 | **<0.001** |
| Mostly remote with some onsite work | 0.88 | 0.74 – 1.04 | **0.124** |
| Fully remote | 0.97 | 0.80 – 1.19 | 0.763 |
| **Work satisfaction** |  |  |  |
| Extremely satisfied | *Reference* |  |  |
| Very satisfied | 0.85 | 0.57 – 1.27 | 0.422 |
| Moderately satisfied | 2.14 | 1.48 – 3.12 | **<0.001** |
| Neither dissatisfied nor satisfied | 5.10 | 3.49 – 7.53 | **<0.001** |
| Moderately dissatisfied | 6.67 | 4.52 – 9.95 | **<0.001** |
| Very dissatisfied | 13.27 | 8.44 – 21.24 | **<0.001** |
| Extremely dissatisfied | 14.12 | 8.55 – 23.90 | **<0.001** |
| **Depression** |  |  |  |
| Normal or mild | *Reference* |  |  |
| Moderate | 6.21 | 5.11 – 7.56 | **<0.001** |
| Severe or extremely severe | 28.42 | 23.67 – 34.25 | **<0.001** |
| **Anxiety** |  |  |  |
| Normal or mild | *Reference* |  |  |
| Moderate | 4.02 | 3.35 – 4.83 | **<0.001** |
| Severe or extremely severe | 14.19 | 12.03 – 16.79 | **<0.001** |
| **Stress** |  |  |  |
| Normal or mild | *Reference* |  |  |
| Moderate | 6.37 | 5.26 – 7.74 | **<0.001** |
| Severe or extremely severe | 24.20 | 19.68 – 30.02 | **<0.001** |

**Table 4.** Association between sociodemographic variables and psychological distress with burnout by country – Malaysia (n=2381)

|  | **Burnout** | | |
| --- | --- | --- | --- |
| *Estimates* | *Odds Ratios* | *95% CIs* | *p-values* |
| **Gender** |  |  |  |
| Male | *Reference* |  |  |
| Female | 1.26 | 0.96 – 1.65 | 0.099 |
| Other | 0.85 | 0.09 – 8.38 | 0.881 |
| **Age** |  |  |  |
| 18-30 | *Reference* |  |  |
| 30-39 | 0.90 | 0.67 – 1.21 | 0.486 |
| 40-49 | 0.76 | 0.49 – 1.17 | 0.209 |
| 50-65 | 0.91 | 0.44 – 1.82 | 0.798 |
| **Relationship status** |  |  |  |
| Single | *Reference* |  |  |
| Casually dating | 0.90 | 0.56 – 1.46 | 0.672 |
| In a long-term relationship | 0.79 | 0.54 – 1.18 | 0.247 |
| Married or in a domestic partnership | 1.15 | 0.85 – 1.54 | 0.367 |
| Divorced, or separated | 1.29 | 0.59 – 2.84 | 0.528 |
| Widowed | 0.27 | 0.03 – 1.95 | 0.200 |
| **Industry** |  |  |  |
| Science and Technology | *Reference* |  |  |
| Education & Training | 1.37 | 0.49 – 3.82 | 0.554 |
| Administration & Office Support | 1.04 | 0.37 – 2.91 | 0.943 |
| Mining, Resources & Energy | 1.31 | 0.35 – 4.87 | 0.688 |
| Manufacturing, Transport & Logistics | 1.00 | 0.35 – 2.81 | 0.995 |
| Accounting | 0.77 | 0.27 – 2.18 | 0.627 |
| Engineering | 0.99 | 0.35 – 2.77 | 0.978 |
| Sales | 1.57 | 0.44 – 5.72 | 0.493 |
| Call Centre & Customer Service | 0.55 | 0.18 – 1.65 | 0.285 |
| Banking & Financial Services | 1.00 | 0.36 – 2.74 | 0.996 |
| Trades & Services | 5.24 | 0.89 – 38.14 | 0.079 |
| Information & Communication Technology | 0.95 | 0.34 – 2.66 | 0.917 |
| Healthcare & Medical | 1.18 | 0.42 – 3.29 | 0.749 |
| Advertising, Arts & Media | 0.55 | 0.18 – 1.67 | 0.288 |
| Retail & Consumer Products | 1.64 | 0.54 – 4.97 | 0.380 |
| Hospitality & Tourism | 2.30 | 0.58 – 9.65 | 0.243 |
| Construction | 1.30 | 0.44 – 3.81 | 0.631 |
| Human Resources & Recruitment | 1.83 | 0.57 – 5.94 | 0.310 |
| Design & Architecture | 0.89 | 0.23 – 3.52 | 0.868 |
| Legal | 0.93 | 0.26 – 3.39 | 0.912 |
| Consulting & Strategy | 1.17 | 0.37 – 3.67 | 0.791 |
| Real Estate & Property | 2.45 | 0.69 – 8.70 | 0.164 |
| Government & Defence | 1.50 | 0.40 – 5.66 | 0.548 |
| Marketing & Communications | 1.46 | 0.49 – 4.34 | 0.496 |
| Community Services & Development | 0.68 | 0.14 – 3.18 | 0.629 |
| Sport & Recreation | 2.29 | 0.10 – 25.41 | 0.528 |
| Insurance & Superannuation | 0.34 | 0.09 – 1.24 | 0.103 |
| Farming, Animals & Conservation | 1.57 | 0.20 – 13.77 | 0.674 |
| Others | 2.06 | 0.71 – 5.93 | 0.181 |
| **Seniority** |  |  |  |
| Entry level | *Reference* |  |  |
| Senior Management | 0.69 | 0.35 – 1.33 | 0.265 |
| Middle Management | 1.16 | 0.73 – 1.84 | 0.532 |
| Lower Management | 1.10 | 0.71 – 1.69 | 0.673 |
| Non-managerial executive | 1.21 | 0.82 – 1.80 | 0.330 |
| Not applicable | 0.87 | 0.46 – 1.66 | 0.672 |
| **Average hours worked per week** |  |  |  |
| 40-50 hours a week | *Reference* |  |  |
| Less than 40 hours/week | 1.30 | 1.01 – 1.67 | **0.045** |
| More than 50 hours/week | 1.59 | 1.10 – 2.31 | **0.014** |
| **Work situation** |  |  |  |
| Fully onsite | *Reference* |  |  |
| Mostly onsite with some remote work | 1.23 | 0.90 – 1.68 | 0.187 |
| Mostly remote with some onsite work | 1.04 | 0.75 – 1.44 | 0.821 |
| Fully remote | 0.99 | 0.63 – 1.56 | 0.964 |
| **Work satisfaction** |  |  |  |
| Extremely satisfied | *Reference* |  |  |
| Very satisfied | 0.67 | 0.31 – 1.47 | 0.311 |
| Moderately satisfied | 2.65 | 1.32 – 5.51 | **0.007** |
| Neither dissatisfied nor satisfied | 5.42 | 2.64 – 11.46 | **<0.001** |
| Moderately dissatisfied | 6.48 | 3.13 – 13.84 | **<0.001** |
| Very dissatisfied | 14.47 | 6.41 – 33.71 | **<0.001** |
| Extremely dissatisfied | 8.90 | 3.49 – 23.76 | **<0.001** |
| **Depression level** |  |  |  |
| Normal or mild | *Reference* |  |  |
| Moderate | 3.23 | 2.38 – 4.38 | **<0.001** |
| Severe or Extremely Severe | 6.71 | 4.73 – 9.57 | **<0.001** |
| **Anxiety level** |  |  |  |
| Normal or mild | *Reference* |  |  |
| Moderate | 2.15 | 1.57 – 2.93 | **<0.001** |
| Severe or Extremely Severe | 2.72 | 1.92 – 3.86 | **<0.001** |
| **Stress level** |  |  |  |
| Normal or mild | *Reference* |  |  |
| Moderate | 1.80 | 1.27 – 2.57 | **0.001** |
| Severe or Extremely Severe | 4.43 | 2.90 – 6.83 | **<0.001** |

Bolded p-values represent p<0.005. McFadden’s adjusted R^2^= 0.385; Cragg-Uhler (Nagelkerke) R^2^= 0.588; Akaike information criterion (AIC) = 1992.128; Hosmer & Lemeshow test χ2 = 18.8523, p<0.05; Multicollinearity checks indicated no multicollinearity between all listed factors (GVIF<5.00).

**Table 5.** Association between sociodemographic variables and psychological distress with burnout by country – Indonesia (n=337)

|  | **Burnout** | | |
| --- | --- | --- | --- |
| *Estimates* | *Odds Ratios* | *95% CIs* | *p-values* |
| **Gender** |  |  |  |
| Male | *Reference* |  |  |
| Female | 0.70 | 0.33 – 1.41 | 0.323 |
| **Age** |  |  |  |
| 18-30 | *Reference* |  |  |
| 30-39 | 0.78 | 0.33 – 1.89 | 0.583 |
| 40-49 | 1.64 | 0.38 – 7.36 | 0.510 |
| *50-65* | 0.00 | NA – 3171492369202714 064424642884020806040 882644826866846662466 8444206206662860806866 8824644046024608200460 6488464080688086266202 24262.00 | 0.993 |
| **Relationship status** |  |  |  |
| Single | *Reference* |  |  |
| Casually dating | 0.44 | 0.17 – 1.16 | 0.094 |
| In a long-term relationship | 0.35 | 0.14 – 0.85 | **0.021** |
| Married or in a domestic partnership | 0.45 | 0.18 – 1.13 | 0.091 |
| Divorced, or separated | 0.09 | 0.00 – 3.12 | 0.123 |
| *Widowed* | 0.00 | NA – 814570729023681 24912248806848684824 64044662848006244480 06640264642466626828 22040248068460000040 46888288686622602026 0242468022486.00 | 0.992 |
| **Seniority** |  |  |  |
| Entry level | *Reference* |  |  |
| Senior Management | 1.31 | 0.30 – 5.91 | 0.719 |
| Middle Management | 0.76 | 0.25 – 2.42 | 0.637 |
| Lower Management | 1.86 | 0.77 – 4.63 | 0.172 |
| Non-managerial executive | 1.03 | 0.45 – 2.32 | 0.949 |
| *Not applicable* | 16.48 | 1.90 – 397.69 | **0.028** |
| **Average hours worked per week** |  |  |  |
| 40-50 hours a week | *Reference* |  |  |
| Less than 40 hours/week | 1.63 | 0.83 – 3.31 | 0.164 |
| More than 50 hours/week | 1.57 | 0.60 – 4.37 | 0.373 |
| **Work situation** |  |  |  |
| Fully onsite | *Reference* |  |  |
| Mostly onsite with some remote work | 1.35 | 0.57 – 3.27 | 0.499 |
| Mostly remote with some onsite work | 0.86 | 0.39 – 1.90 | 0.703 |
| Fully remote | 0.91 | 0.28 – 3.14 | 0.876 |
| **Work satisfaction** |  |  |  |
| Extremely satisfied | *Reference* |  |  |
| Very satisfied | 1.76 | 0.12 – 25.56 | 0.674 |
| Moderately satisfied | 5.47 | 0.41 – 76.10 | 0.194 |
| Neither dissatisfied nor satisfied | 8.55 | 0.62 – 122.74 | 0.105 |
| Moderately dissatisfied | 12.89 | 0.91 – 188.89 | 0.056 |
| Very dissatisfied | 12.22 | 0.80 – 200.01 | 0.070 |
| Extremely dissatisfied | 6.73 | 0.42 – 111.48 | 0.174 |
| **Depression level** |  |  |  |
| Normal or mild | *Reference* |  |  |
| Moderate | 3.87 | 1.57 – 9.86 | **0.004** |
| Severe or Extremely Severe | 5.76 | 2.35 – 14.58 | **<0.001** |
| **Anxiety level** |  |  |  |
| Normal or mild | *Reference* |  |  |
| Moderate | 2.35 | 0.97 – 5.80 | 0.059 |
| Severe or Extremely Severe | 2.64 | 1.07 – 6.60 | **0.036** |
| **Stress level** |  |  |  |
| Normal or mild | *Reference* |  |  |
| Moderate | 2.79 | 1.19 – 6.71 | **0.019** |
| Severe or Extremely Severe | 6.22 | 2.42 – 16.55 | **<0.001** |

Bolded p-values represent p<0.005. McFadden’s adjusted R^2^= 0.230; Cragg-Uhler (Nagelkerke) R^2^= 0.531; Akaike information criterion (AIC) = 339.801; Hosmer & Lemeshow test χ2 = 9.044, p>0.05; Multicollinearity checks indicated no multicollinearity between all listed factors (GVIF<5.00).

**Table 6.** Association between sociodemographic variables and psychological distress with burnout by country – Singapore (n=401)

|  | **Burnout** | | |
| --- | --- | --- | --- |
| *Estimates* | *Odds Ratios* | *95% CIs* | *p-values* |
| **Gender** |  |  |  |
| Male | *Reference* |  |  |
| Female | 1.32 | 0.56 – 3.14 | 0.519 |
| **Age** |  |  |  |
| 18-30 | *Reference* |  |  |
| 30-39 | 0.68 | 0.27 – 1.69 | 0.404 |
| 40-49 | 1.20 | 0.37 – 4.07 | 0.762 |
| 50-65 | 0.08 | 0.00 – 1.16 | 0.107 |
| **Relationship status** |  |  |  |
| Single | *Reference* |  |  |
| Casually dating | 1.66 | 0.39 – 8.09 | 0.511 |
| In a long-term relationship | 0.90 | 0.33 – 2.47 | 0.834 |
| Married or in a domestic partnership | 1.64 | 0.62 – 4.42 | 0.319 |
| Divorced, or separated | 1.38 | 0.08 – 22.06 | 0.822 |
| *Widowed* | 0.00 | NA – ∞ | 0.999 |
| **Industry** |  |  |  |
| Science and Technology | *Reference* |  |  |
| Education & Training | 0.64 | 0.02 – 8.01 | 0.758 |
| Administration & Office Support | 0.23 | 0.01 – 3.36 | 0.338 |
| *Mining, Resources & Energy* | 0.00 | NA – ∞ | 0.998 |
| Manufacturing, Transport & Logistics | 0.05 | 0.00 – 0.83 | 0.059 |
| Accounting | 0.21 | 0.01 – 3.84 | 0.337 |
| Engineering | 1.17 | 0.04 – 15.99 | 0.913 |
| Sales | 0.40 | 0.01 – 15.59 | 0.636 |
| Call Centre & Customer Service | 2.99 | 0.07 – 86.15 | 0.530 |
| Banking & Financial Services | 0.36 | 0.01 – 5.20 | 0.498 |
| Trades & Services | 0.14 | 0.00 – 7.96 | 0.346 |
| Information & Communication Technology | 1.53 | 0.04 – 32.93 | 0.798 |
| Healthcare & Medical | 1.36 | 0.04 – 18.62 | 0.837 |
| Advertising, Arts & Media | 0.11 | 0.00 – 2.55 | 0.187 |
| Retail & Consumer Products | 0.07 | 0.00 – 1.19 | 0.098 |
| Hospitality & Tourism | 0.97 | 0.02 – 22.75 | 0.985 |
| Construction | 0.15 | 0.00 – 2.35 | 0.224 |
| Human Resources & Recruitment | 0.84 | 0.02 – 19.31 | 0.917 |
| Design & Architecture | 1.25 | 0.02 – 43.26 | 0.904 |
| *Legal* | 78336551.60 | 0.00 – ∞ | 0.996 |
| Consulting & Strategy | 1.14 | 0.02 – 41.32 | 0.943 |
| Real Estate & Property | 0.07 | 0.00 – 14.21 | 0.365 |
| Government & Defence | 4.68 | 0.12 – 110.47 | 0.355 |
| Marketing & Communications | 5.86 | 0.11 – 268.75 | 0.338 |
| *Community Services & Development* | 24768818.56 | 0.00 – ∞ | 0.998 |
| Sport & Recreation | 0.14 | 0.00 – 20.03 | 0.534 |
| Insurance & Superannuation | 2.57 | 0.04 – 138.39 | 0.637 |
| *Farming, Animals & Conservation* | 6433572.63 | 0.00 – ∞ | 0.998 |
| Others | 0.13 | 0.00 – 1.89 | 0.179 |
| **Seniority** |  |  |  |
| Entry level | *Reference* |  |  |
| Senior Management | 1.15 | 0.16 – 7.45 | 0.887 |
| Middle Management | 0.86 | 0.22 – 3.30 | 0.819 |
| Lower Management | 0.35 | 0.09 – 1.31 | 0.120 |
| Non-managerial executive | 0.92 | 0.29 – 2.89 | 0.881 |
| Not applicable | 2.17 | 0.35 – 16.91 | 0.428 |
| **Average hours worked per week** |  |  |  |
| 40-50 hours a week | *Reference* |  |  |
| Less than 40 hours/week | 0.70 | 0.29 – 1.65 | 0.415 |
| More than 50 hours/week | 1.40 | 0.43 – 4.69 | 0.579 |
| **Work situation** |  |  |  |
| Fully onsite | *Reference* |  |  |
| Mostly onsite with some remote work | 0.81 | 0.32 – 2.03 | 0.643 |
| Mostly remote with some onsite work | 2.26 | 0.75 – 7.06 | 0.150 |
| Fully remote | 0.21 | 0.03 – 1.39 | 0.103 |
| **Work satisfaction** |  |  |  |
| Extremely satisfied | *Reference* |  |  |
| Very satisfied | 1.20 | 0.16 – 9.31 | 0.862 |
| Moderately satisfied | 1.90 | 0.38 – 10.58 | 0.444 |
| Neither dissatisfied nor satisfied | 2.73 | 0.52 – 16.13 | 0.245 |
| Moderately dissatisfied | 7.85 | 1.43 – 49.53 | **0.021** |
| *Very dissatisfied* | 926052225.66 | 0.00 – 1146742 2151044698009 60008406860668 66828662682222 48086806446022 88088664626420 84462488664440 22848286086262 64448482064424 408606248624428 488864026446826 802624286466042 680884824600668 206626866802608 040640248400488 24446.00 | 0.987 |
| *Extremely dissatisfied* | 14.86 | 1.85 – 143.39 | **0.014** |
| **Depression level** |  |  |  |
| Normal or mild | *Reference* |  |  |
| Moderate | 1.82 | 0.62 – 5.40 | 0.276 |
| Severe or Extremely Severe | 12.04 | 4.15 – 37.73 | **<0.001** |
| **Anxiety level** |  |  |  |
| Normal or mild | *Reference* |  |  |
| Moderate | 2.86 | 1.13 – 7.44 | **0.028** |
| Severe or Extremely Severe | 3.38 | 1.19 – 9.81 | **0.023** |
| **Stress level** |  |  |  |
| Normal or mild | *Reference* |  |  |
| Moderate | 2.18 | 0.83 – 5.85 | 0.115 |
| Severe or Extremely Severe | 6.15 | 1.99 – 20.53 | **0.002** |

Bolded p-values represent p<0.005. McFadden’s adjusted R^2^= 0.305; Cragg-Uhler (Nagelkerke) R^2^= 0.691; Akaike information criterion (AIC) = 354.022; Hosmer & Lemeshow test χ2 = 12.96, p>0.05; Multicollinearity checks indicated no multicollinearity between all listed factors (GVIF<5.00).

**Table 7.** Association between sociodemographic variables and psychological distress with burnout by country – Philippines (n=1219)

|  | **Burnout** | | |
| --- | --- | --- | --- |
| *Estimates* | *Odds Ratios* | *95% CIs* | *p-values* |
| **Gender** |  |  |  |
| Male | *Reference* |  |  |
| Female | 1.26 | 0.80 – 2.00 | 0.318 |
| Other | 3.87 | 0.42 – 53.96 | 0.265 |
| **Age** |  |  |  |
| 18-30 | *Reference* |  |  |
| 30-39 | 0.67 | 0.43 – 1.03 | 0.065 |
| 40-49 | 1.02 | 0.44 – 2.38 | 0.962 |
| 50-65 | 1.20 | 0.26 – 5.40 | 0.812 |
| **Relationship status** |  |  |  |
| Single | *Reference* |  |  |
| Casually dating | 0.73 | 0.36 – 1.49 | 0.374 |
| In a long-term relationship | 0.68 | 0.42 – 1.09 | 0.107 |
| Married or in a domestic partnership | 0.68 | 0.40 – 1.14 | 0.143 |
| Divorced, or separated | 0.50 | 0.15 – 1.88 | 0.282 |
| *Widowed* | 7911377.39 | 0.00 – NA | 0.981 |
| **Industry** |  |  |  |
| Science and Technology | *Reference* |  |  |
| Education & Training | 0.47 | 0.05 – 2.93 | 0.454 |
| Administration & Office Support | 0.91 | 0.09 – 5.94 | 0.925 |
| Mining, Resources & Energy | 0.43 | 0.03 – 6.15 | 0.536 |
| Manufacturing, Transport & Logistics | 0.80 | 0.07 – 6.64 | 0.840 |
| Accounting | 1.44 | 0.14 – 9.96 | 0.732 |
| Engineering | 0.83 | 0.07 – 7.33 | 0.877 |
| Sales | 0.68 | 0.06 – 5.26 | 0.723 |
| Call Centre & Customer Service | 0.74 | 0.08 – 4.44 | 0.764 |
| Banking & Financial Services | 1.02 | 0.10 – 7.30 | 0.988 |
| Trades & Services | 0.05 | 0.00 – 4.83 | 0.205 |
| Information & Communication Technology | 0.51 | 0.05 – 3.32 | 0.515 |
| Healthcare & Medical | 0.59 | 0.06 – 3.81 | 0.609 |
| Advertising, Arts & Media | 0.29 | 0.02 – 2.54 | 0.283 |
| Retail & Consumer Products | 1.75 | 0.12 – 20.26 | 0.661 |
| Hospitality & Tourism | 1.03 | 0.09 – 8.69 | 0.983 |
| Construction | 0.50 | 0.04 – 4.07 | 0.543 |
| Human Resources & Recruitment | 1.60 | 0.14 – 13.56 | 0.681 |
| Design & Architecture | 0.41 | 0.03 – 5.08 | 0.502 |
| Legal | 0.39 | 0.03 – 5.49 | 0.480 |
| Consulting & Strategy | 0.87 | 0.07 – 8.22 | 0.910 |
| Real Estate & Property | 0.78 | 0.05 – 10.13 | 0.850 |
| Government & Defence | 0.52 | 0.05 – 3.77 | 0.544 |
| Marketing & Communications | 0.39 | 0.03 – 4.24 | 0.450 |
| Community Services & Development | 0.49 | 0.02 – 8.26 | 0.632 |
| *Sport & Recreation* | 0.30 | 0.00 – 53.24 | 0.715 |
| *Insurance & Superannuation* | 1.51 | 0.04 – 69.06 | 0.820 |
| *Farming, Animals & Conservation* | 580370.45 | 24855677679885 62734828480882 04604402668024 84626466868686 68820404684660 66624066806466 88284446448844 06282420422006 60840884684808 80402280224080 40440400260688 66460066828440 28842444440660 26004066246040 8284020.00 –  102236563994411 96531208042020 06806286622000 00604848204844 28884286482888 62062442002028 86806040468862 02808800040422 68224446226484 8248444002.00 | 0.985 |
| Others | 1.00 | 0.09 – 7.57 | 0.999 |
| **Seniority** |  |  |  |
| Entry level | *Reference* |  |  |
| Senior Management | 2.64 | 0.83 – 8.65 | 0.103 |
| Middle Management | 1.55 | 0.79 – 3.07 | 0.205 |
| Lower Management | 1.48 | 0.87 – 2.55 | 0.150 |
| Non-managerial executive | 1.02 | 0.63 – 1.65 | 0.934 |
| Not applicable | 1.26 | 0.55 – 2.94 | 0.588 |
| **Work situation** |  |  |  |
| Fully onsite | *Reference* |  |  |
| Mostly onsite with some remote work | 0.82 | 0.43 – 1.57 | 0.550 |
| Mostly remote with some onsite work | 0.93 | 0.52 – 1.69 | 0.814 |
| Fully remote | 0.88 | 0.54 – 1.44 | 0.603 |
| **Work satisfaction** |  |  |  |
| Extremely satisfied | *Reference* |  |  |
| Very satisfied | 2.02 | 0.76 – 5.35 | 0.157 |
| Moderately satisfied | 5.08 | 2.00 – 12.80 | **0.001** |
| Neither dissatisfied nor satisfied | 6.18 | 2.34 – 16.17 | **<0.001** |
| Moderately dissatisfied | 11.66 | 4.21 – 32.46 | **<0.001** |
| Very dissatisfied | 30.49 | 8.58 – 116.64 | **<0.001** |
| Extremely dissatisfied | 14.89 | 3.84 – 65.56 | **<0.001** |
| **Depression level** |  |  |  |
| Normal or mild | *Reference* |  |  |
| Moderate | 3.60 | 2.13 – 6.17 | **<0.001** |
| Severe or Extremely Severe | 8.06 | 4.75 – 13.85 | **<0.001** |
| **Anxiety level** |  |  |  |
| Normal or mild | *Reference* |  |  |
| Moderate | 1.78 | 1.03 – 3.08 | **0.040** |
| Severe or Extremely Severe | 1.62 | 0.98 – 2.68 | 0.059 |
| **Stress level** |  |  |  |
| Normal or mild | *Reference* |  |  |
| Moderate | 3.05 | 1.88 – 5.01 | **<0.001** |
| Severe or Extremely Severe | 9.87 | 5.62 – 17.71 | **<0.001** |

Bolded p-values represent p<0.005. McFadden’s adjusted R^2^= 0.372; Cragg-Uhler (Nagelkerke) R^2^= 0.601; Akaike information criterion (AIC) = 925.181; Hosmer & Lemeshow test χ2 = 7.80, p>0.05; Multicollinearity checks indicated no multicollinearity between all listed factors (GVIF<5.00).
